# Case report: Goiter and overt hypothyroidism in an iodine-deficient toddler on soy milk and hypoallergenic diet

**DOI:** 10.3389/fendo.2022.927726

**Published:** 2022-08-11

**Authors:** Angela Maria Caprio, Giuseppina Rosaria Umano, Caterina Luongo, Francesca Aiello, Iride Dello Iacono, Stefania Palumbo, Emanuele Miraglia del Giudice, Anna Grandone

**Affiliations:** ^1^ Department of the Woman, The Child, of General and Specialized Surgery, University of Campania “Luigi Vanvitelli”, Naples, Italy; ^2^ Unit of Allergology. Division of Internal Medicine, Fatebenefratelli Hospital, Benevento, Italy

**Keywords:** children, goiter, hypothyroidism, soybean, cow’s milk allergy, case report

## Abstract

Soy-based infant formulas (SFs) are often consumed by cow’s milk allergic children. However, some concerns have risen since soy intake may adversely affect thyroid function in iodine-deficient or subclinical hypothyroid individuals. We report the first Italian case of SF induced goiter and hypothyroidism registered in our country since National Iodine program has been instituted. Finally, we review cases previously reported in literature. A 22-month-old toddler with a previous diagnosis of cow’s milk protein allergy came to clinical attention for important goiter and overt hypothyroidism. Detailed dietary anamnesis revealed that he was on a restrictive dietary regimen based on soymilk since 12 months of age. A temporary levothyroxine substitution was instituted to avoid hypothyroidism complications. Adequate iodine supplementation and diet diversification completely reversed SF-induced hypothyroidism and goiter, confirming the diagnostic suspicion of soymilk-induced thyroid dysfunction in a iodine-deficient toddler. This case report demonstrates the importance of careful dietary habits investigation and adequate micronutrients supplementation in children on a restrictive diet due to multiple food allergies in order to prevent nutritional deficits.

## Introduction

Soy-based infant formula (SF) has been massively used as an alternative diet in children with cow’s milk allergy or lactose intolerance (1). However, in the early to mid-1900s, the discovery of a possible thyreotoxic effect of phyto-estrogenic isoflavonoids contained in soybean has risen some concerns about SF use in infants. Isoflavonoids inhibit thyroid peroxidase (TPO) by acting as alternative substrates for iodination (2–4). By contrast, later studies demonstrated that this effect becomes clinically relevant only when iodine intake is insufficient (5, 6). A systematic review in 2007 reported that modern iodine-enriched SFs adequately support growth and development. Indeed, no case of goiter and/or hypothyroidism has been described with iodine-enriched SFs. The combined effect of soy foods consumption and iodine deficiency on thyroid volume and function was later confirmed in rodents models (2, 7, 8).

Here, we reported the case of a 22-month-old toddler who was put on a soy-based diet due to cow’s milk protein allergy and developed goiter and overt hypothyroidism. This is the very first case in Italian population and, to the best of our knowledge, a unique report in countries where the Iodine Deficiency Disorders Control Programme is ongoing. We retrospectively analyzed other case reports in humans and synthesized available data on thyroid dysfunction risk associated with SF.

## Case presentation

A 22-month-old Italian male infant presented at the outpatient Pediatric Endocrinological service of the A.O.U. “Luigi Vanvitelli” with a 3-month history of progressive anterior neck swelling. All clinical data, in here presented, and related images have been disclosed in accordance with the Helsinki declaration, after both parents gave informed consent to publication and data were anonymized.

Familial history was relevant for autoimmune-thyroiditis-induced hypothyroidism in the mother. Mid-parental height was 177 cm (+0.07 SDS). The baby was the only child of unrelated parents. He was born full-term from an uncomplicated pregnancy. At birth, he weighted 3.660 kg (+1.21 SDS according to Neonatal Anthropometric Charts for the Italian population, Bertino et al. 2010), length was 50 cm (+0.26 SDS), and head circumference was 35 cm (+0.69 SDS). The mother’s thyroid function was normal during pregnancy and received no drug affecting thyroid function. The baby passed neonatal screening for congenital hypothyroidism. He had been formula fed since birth. Clinical history revealed normal psico-motor development. At 4 months of age, he suffered from food-protein-induced enterocolitis syndrome; since then, he followed a cow’s milk protein-free diet. Hence, the child only received extensively hydrolyzed formula until 1 year of age. Then, he was switched to soy-based infant formula. The weaning process was never completed due to his parents’ fear of food allergy reactions. At 22 months of age, the baby diet was very selective: soymilk represented the main nutritional source (with a daily intake of 800–1,000 ml) associated with small amount of carbohydrates (rice or pasta). No protein-rich foods (fish, meat, and eggs) were provided. Moreover, he had a low-salt diet; therefore, iodine intake appeared inadequate.

**Figure 1:**
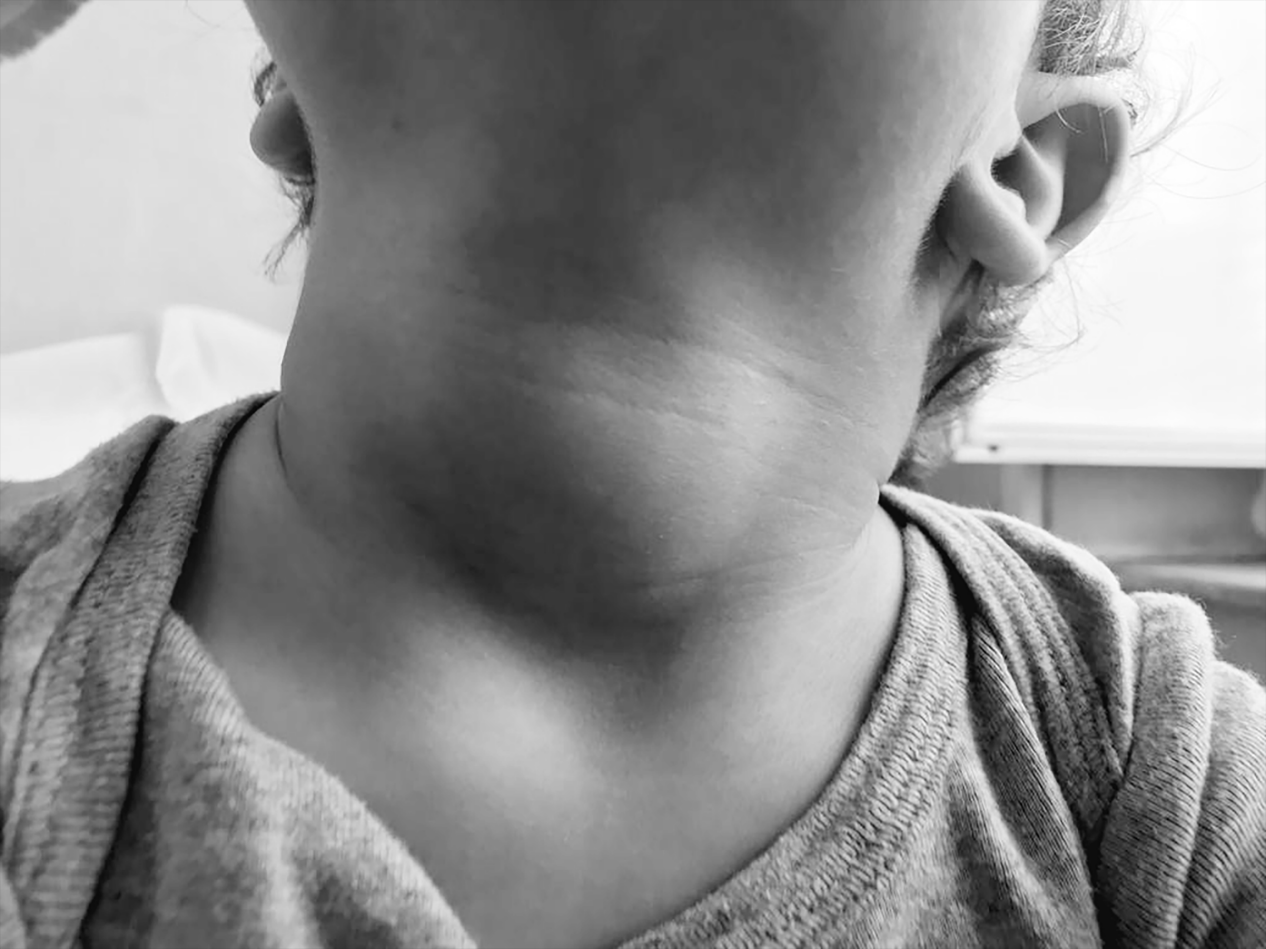
Child’s neck at diagnosis: thyroid gland was considerably increased in size, soft, non tender and freely movable with swallowing.

On physical examination, auxological parameters were within normal according to the WHO growth chart, 2006: he weighed 10.650 kg (−0.88 SDS), his height was 83.5 cm (−0.87 SDS), and his head circumference measured 48 cm (+0.79 SDS). Neck palpation revealed a soft, non-tender, diffuse swelling of the thyroid gland (shown in **Figure 1**). He showed dry skin, thin hair, and delayed teeth eruption.

## Investigation and management

Thyroid ultrasound confirmed thyromegaly: gland volume measured 8.7 ml (> +1 SD according to age and sex reference values) with no underlying mass. Laboratory testing showed elevated serum thyrotropin concentration [thyroid-stimulating hormone (TSH), 47.510 µU/ml; normal range, 0.1–4.1], low serum-free tetraiodothyronin (FT4, 1 pg/ml; reference value, 4.4–12 pg/ml), and normal free triiodothyronine (FT3, 3.7 pg/ml; reference value, 1.0–4.3 pg/ml). Serum thyroglobulin was increased (TG, 701.5 ng/ml; reference, <60 ng/ml). Thyroid antibodies were not detected. Taking into account the importance of proper thyroid function in the first years of life, the baby was placed at levothyroxine replacement therapy (about 1 µg/kg/die).

Differential diagnoses of SF-induced goiter in iodine-deficient toddlers and congenital hypothyroidism were considered. As the baby presented normal neonatal thyroid screening, a normal thyroid volume, and no symptoms of hypothyroidism in the first year of life, congenital hypothyroidism does not seem likely. Thus, there was a strong suspicion of iodine deficiency, as pointed out by dietary anamnesis. Therefore, we first estimated iodine intake, analyzing urinary iodine output from a 24-h urine sample collection. Urinary iodine concentration (UIC) was strongly reduced (<8 µg/L). According to current FAO/WHO daily recommended nutrient intake for iodine, we prescribed a dietary salt intake of 3 g containing about 90 µg of iodine per day (9). Moreover, soymilk was replaced with extensive hydrolyzed formula. Fish, meat, eggs, and fruit were gradually introduced into his diet.

For the sake of completeness, genetic testing was performed in order to rule out congenital hypothyroidism due to thyroid peroxidase (TPO) deficiency. Genomic DNA of our patient was extracted from peripheral whole blood sample, using a DNA extraction kit (Promega, Madison WI, USA). A direct sequencing of *TPO* gene was performed by Sanger method under standard conditions. PCR analysis found no mutation.

The final diagnosis was secondary hypothyroidism due to iodine deficiency, complicated by soymilk consumption.

## Outcomes

**Figure 2:**
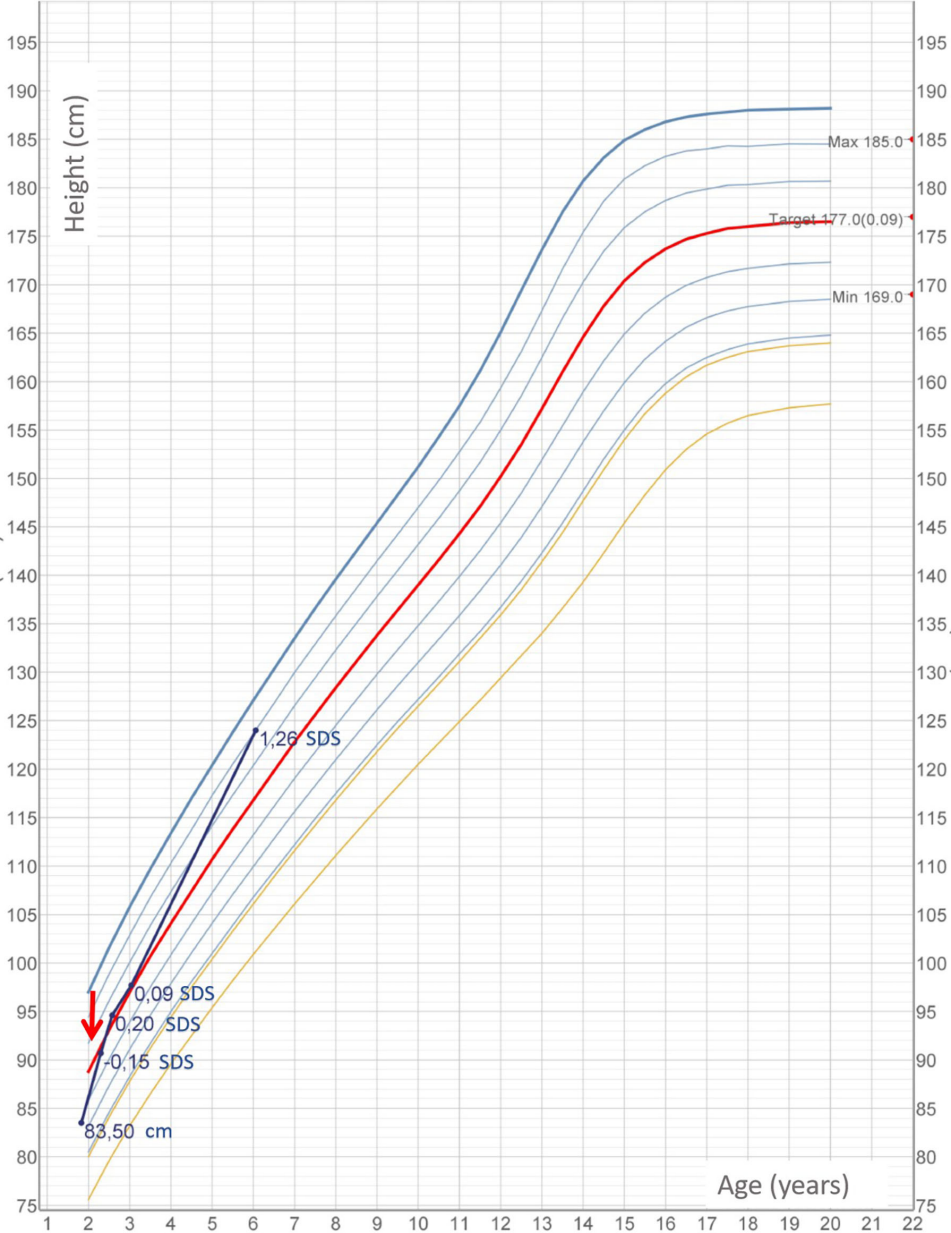
Child’s height measurements according to Cacciari growth chart 2006 for Italian population. Red arrow marks beginning of euthyroid status.

Within 3 weeks since the beginning of levothyroxine treatment, thyroid function normalized (TSH, 3.38 mU/L; FT4, 10 ng/ml). Thyroglobulin decreased (138 ng/ml), although not within normal range. Gland size remarkably reduced. After 3 months of supplementation, patient showed increased growth rate, good general conditions, and reduced thyroid volume. Levothyroxine was tapered within 3 months of treatment and stopped at 25 months of age. In the first year of follow-up, thyroglobulin and median UIC completely normalized. At the age of 3.5 years, the baby maintained in the euthyroid state, and thyroid gland was no longer palpable. Auxological parameters were normal [height, 97.7cm: +0.58 SDS; weight, 14 kg; and body mass index (BMI), 16.5 kg/m^2^]. At the age of 6.25 years, height was 124 cm, +1.3 SDS; he weighed 21.2, and his BMI was 18.6 kg/m^2^. Thyroid function was normal (TSH, 0.937 µU/ml, FT4, 10.2 pg/ml). The growth chart is shown in **Figure 2**.

## Discussion

This case is unique because the timeline of events suggests that severe biochemical hypothyroidism and goiter can be triggered by SF in children on very selective diet. To our knowledge, this is the first case report of soy-induced goiter in countries where an iodization program is ongoing. In Italy, national iodine prophylaxis has been introduced since 2005 with the approval of Law 55/2005, which regulates the sale and use of iodized salt (iodine content, 30 mg/kg of salt). Periodic reports by the National Observatory for the Monitoring of Iodine Prophylaxis in Italy (OSNAMI) of the ISS demonstrated that Italy achieved iodine sufficiency since 2015 (10).

Etiopathogenesis of soy-induced goiter and overt hypothyroidism has been widely investigated in the literature. Soy isoflavonoids (genistein and daidzein) act as goitrogenic substances by the following mechanisms: (a) inhibiting the iodination activity of TPO and (b) increasing hormonal iodine fecal losses. However, animal and human studies data point out that soy-induced overt hypothyroidism and goiter are uncommon in the absence of other risk factors. Ikeda et al. (2) demonstrated, similarly to Kimura et al. (7), that feeding rats a soy-containing diet caused severe hypothyroidism only when iodine deficiency was present. In 2017, a recent systematic review carried out in the general population revealed that euthyroid individuals with adequate iodine intake had modest isolated TSH rise with no significant changes in FT3 and FT4 levels when exposed to soy isoflavonoids (6). By contrast, in the presence of subclinical hypothyroidism or risk factors for thyroid dysfunction, such as iodine deficiency, as in our patient, soy isoflavonoids can produce clinically significant effects.

We performed a review of all cases of soy-induced goiter and/or hypothyroidism recorded in the literature. We search in PubMed database and Google scholar with terms: [“children”AND/OR”infant”] AND [“soybean”OR/AND”soy-milk”OR/AND”soy-based formula”] AND [“goiter”AND/OR”thyroid dysfunction”]. Results focused on case reports published in English. A total of six reported cases was identified; no case has been reported after 1995. The presentation and management of patients are summarized in **Tables 1** **,** **2**. Two reports pointed out the risk of interference of SF with levothyroxine substitutive therapy in congenital hypothyroidism (see papers 5 and 6). In these cases, the initial increase in medium therapy dosage partially addressed hypothyroidism, while whole cow’s milk substitution totally stabilized the euthyroid state and prevented from further therapy dosage increases. The remaining cases shared in common no signs of previous thyroid impairment before soy intake, familial or personal history of protein allergy, and no clinical sign of hypothyroidism. Intervention for goiter correction varies among cases, from SF stopping to iodine addition to diet.

**Table 1:** Laboratory findings during follow-up. Abbreviations: free Thyroxine(fT4), Thyroid.

**Age months**	**TSHμ UI/ml**	**FT4 pg/mL**	**Tg µg/L**	**UIC µg/L**	**THERAPY**(Levothyroxine)
22 (diagnosis)	47.5	1	701	<8	Start therapy1 μg/kg/die
22	3.38	10	138		1 μg/kg/die
23	1.509	13.2	97.43	70	0.5 μg/kg/die
24	1.85	10	71.24		Stop therapy
27	0.9	13.2	54.17	>100	no therapy
30	0.8	11.4	56.09		no therapy
36	0.8	9.7	45.01		no therapy
42	0.9	11.1	38		no therapy
75	0.94	10.2	31.21		

Stimulating Hormone (TSH), Urinary iodine concentration (UIC) and Thyroglobulin (Tg).

**Table 2:** Literature review of presentation and management of patients.

	Author Year of publication	Journal	Age and gender	Duration of soymilk formula consumption	Clinical examination	Intervention
1 (11)	Hydovitz JD.1960,	N Engl J Med	5 months Male	4 months	Diffuse thyromegalia of rubbery consistency. Normal growth pattern. No clinical signs of hypothyroidism.	Cow’s milk substitution with remission in one month.
2 (12)	Shepard TH 1960 United States	N Engl J Med	10 months Female 3 months Female 39 months Female	6 months Since birth 36 months due to eczema experienced at 3 months of age	Goiter, but euthyroid Goiter, but euthyroid Goiter, but euthyroid	Soybean formula discontinuation at 11 months of age with total remission in 3 month and half Cow’s milk substitution with remission after 1.5 months. Q[CE] Please provide the exact mountain Lugol’s solution with remission after 15 days.
3 (13)	Ripp JW. 1961 United States	Am J Dis Child	15.5 months Male	12 months since he experienced a severe eczema and spitting at 3.5 months.	Diffuse soft swelling of all lobes of the thyroid gland. Normal growth pattern. no clinical signs of hypothyroidism.	Goat’s milk was substituted for soybean milk with rapid remission. After, total recovery, at 21.5 months of age, a trial with soy-milk for 80 days, demonstrated to cause slight thyroid volume increase.
4 (14)	Van Wyk JJ 1959 United States	Pediatrics	8 months Female,	Since birth due to a family history of strong allergies until 2 week prior first visit	Diffuse thyromegalia and sign of hypothyroidism (puffy face, marked pallor, thick and protruding tongue, peripheral mottling and carotenoid complexion) Length deficiency NO mental retardation	Lugol’s solution and replacement of the soybean product by whole cow’s milk. Total remission in 14 months
5 (9)	Pinchera A 1965 United States	N Engl J Med	6 months and 3 weeks Male Congenital hypothyroidism on substitutive treatment since 3 months and half	7 week after milk allergy symptoms appeared at 5 months of age and necessity to increase thyroxine therapy	Athyreotic with partial clinical sign of hypothyroidism (puffy face, dry and mottled skin, pallor, irritability, alert). Reduced lenght growth.	replacement of the soybean product by whole cow’s milk with rapid recovery
6 (15)	Chorazy PA et al. 1995 United States	Pediatrics	1 month and 19 days Male Congenital hypothyroidism on substitutive treatment since day 11 of life	Since birth due to family history of cow’s milk intolerance	Difficulty in normalizing thyroid tests. Increased stool frequency	Whole-cow milk diet restored and adjustment of levothyroxine therapy dosage for body weight

Our case report partially differs from others previously reported, as our patient developed an overt hypothyroidism and temporarily needed levothyroxine supplementation in addition to long-term dietary modifications. Age of goiter and hypothyroidism onset, 22 months old, was older than previously reported in the literature. Delayed onset, in our patient could be explained by partial iodine substitution within the first year of life due to specific infant formula, which are iodine fortified.

The clinical history of this child supports the previous evidence of synergism between excess in soybean intake and iodine deficiency to induce goiter and hypothyroidism (2–4). After SF was eliminated and a varied and balanced diet was introduced, euthyroidism was achieved and goiter completely reverted. In addition, genetic analysis performed on *TPO* gene ruled out any possible mutation encoding a partially activated TPO protein that could partially explain or contribute to our child phenotype.

Although a recent review in 2018, investigating global phytoestrogens effects on growing child found soymilk formula are not associated with relevant abnormalities (16), no specific investigation has been carried out on the effects of restrictive soymilk diet on thyroid function in otherwise healthy children. That limits the indications of SF use in infant nutrition. According to the American Academy of Pediatrics (17) and the European Society for Pediatric Gastroenterology, Hepatology, and Nutrition (18), SF has a specific indication for full-term infants suffering from galactosemia and hereditary lactase deficiency. In case of cow’s milk protein allergies, as in our case, different nutritional approaches are preferred. However, it is not unusual in clinical practice to use SF in multiallergic patients as ESPGHAN recent guidelines for cow’s milk protein allergy identify soymilk as a possible second option in babies younger than 6 months who do not tolerate extensively hydrolyzed infant formulas (19).

That case highlights the importance of regularly assessing micro-nutrients in children by visiting a registered dietitian following elimination diets due to multiple food allergies. A detailed nutritional anamnesis can help specialist to detect patients at risk of iodine deficiency. Unfortunately, no specific diagnostic tool has been validated to assess individual iodine intake. In the case herein presented, assessment of UIC on 24-h urinary collection has been performed to gain some information on the iodine intake for the past few days. We acknowledged that UIC cutoffs have been developed to assess population iodine intake and is not recommended as a diagnostic tool for iodine deficiency in a given individual, as high intra-individual day-to-day variability exists (20). However, considering that UIC reflects iodine intake shortly before sampling and variability is due to different natural iodine content and bio-availability of food items, mean iodine levels are presumably constant over days in the case of very restrictive eating habits. Thus, we can reasonably consider that a UIC <100 μg/L in our child pointed out iodine deficiency status. Moreover, circulating thyroglobulin levels were increased in accordance to the correlation existing on a population level between this specific thyroid marker and iodine deficiency (16). In the case herein presented, the high thyroglobulin values and the low levels of UIC reflect the deep and protracted iodine deficiency due to the lack of adequate complementary nutrition and worsened by large consumption of soy-based formula.

Iodine fortification of allergy formulas may be insufficient in some cases, and the risk of iodine deficiency with selective diets regimens increases over time, so an additional iodine supplementation should be tailored on a single patient.

## Conclusions

Despite significant improvements in iodine status of populations worldwide, iodine deficiency continues to be a possible cause of goiter and/or hypothyroidism, especially in multiple food allergic infants on restrictive diet. Particular attention should be paid to the eating habits of these children in order to prevent dangerous nutritional deficits, including iodine deficiency. In addition, some foods can have a negative effect on thyroid function. In conclusion all children, especially children fed by soy formula, should receive complementary feeding with an adequate iodine content, in order to maintain a regular thyroid function.

## Data availability statement

The original contributions presented in the study are included in the article. Further inquiries can be directed to the corresponding author.

## Ethics statement

This study was reviewed and approved by University of Campania Luigi Vanvitelli. Written informed consent to participate in this study was provided by the participants’ legal guardian/next of kin.

## Author contributions

AC and GU prepared the original draft manuscript. FA and GU review and edited the manuscript. CL managed the hypothyroidism. II was responsible of food allergy management. SP performed genetic testing. EG and AG supervised the writing of the manuscript. All authors contributed to the article and approved the submitted version.

## Funding

The publication of this work has been supported by a grant (n° 390) funded by "VALERE:VAnviteLli pEr la RicErca" program of University of Campania "L. Vanvitelli".

## Acknowledgments

We acknowledged the patient’s family for sharing their clinical data for research and science improvement.

## Conflict of interest

The authors declare that the present case report was published in the absence of any commercial or financial relationships that could be construed as a potential conflict of interest.

## Publisher’s note

All claims expressed in this article are solely those of the authors and do not necessarily represent those of their affiliated organizations, or those of the publisher, the editors and the reviewers. Any product that may be evaluated in this article, or claim that may be made by its manufacturer, is not guaranteed or endorsed by the publisher.
